# Efficacy of expressive helping in adult hematologic cancer patients undergoing stem cell transplant: protocol for the Writing for Insight, Strength, and Ease (WISE) study’s two-arm randomized controlled trial

**DOI:** 10.1186/s13063-021-05676-w

**Published:** 2021-10-20

**Authors:** Lauren Whitmore, Taylor Schulte, Katrin Bovbjerg, Madison Hartstein, Jane Austin, George Luta, Lily McFarland, Scott D. Rowley, Themba Nyirenda, Marquita Lewis-Thames, Annette L. Stanton, Heiddis Valdimarsdottir, Kristi Graves, Christine Rini

**Affiliations:** 1 John Theurer Cancer Center, Hackensack University Medical Center, Hackensack, NJ USA; 2 Lombardi Comprehensive Cancer Center, Georgetown University, Washington, DC USA; 3 Department of Medical Social Sciences, Feinberg School of Medicine and the Robert H. Lurie Comprehensive Cancer Center, Northwestern University, Chicago, IL USA; 4 Department of Psychology, William Paterson University, Wayne, NJ USA; 5 Center for Community Health, Feinberg School of Medicine, Northwestern University, Chicago, IL USA; 6 Departments of Psychology and Psychiatry/Biobehavioral Sciences and the Jonsson Comprehensive Cancer Center, University of California, Los Angeles, CA USA; 7 Department of Psychology, Reykjavik University, Reykjavik, Iceland; 8 Department of Oncological Sciences, Icahn School of Medicine at Sinai School, New York, NY USA

**Keywords:** Hematopoietic stem cell transplant, Psychological intervention, Patient-reported outcomes, Randomized controlled trial, Peer support, Expressive writing, Cancer survivors

## Abstract

**Background:**

During, shortly after, and sometimes for years after hematopoietic stem cell transplant, a large proportion of hematological cancer patients undergoing transplant report significant physical and psychological symptoms and reduced health-related quality of life. To address these survivorship problems, we developed a low-burden, brief psychological intervention called expressive helping that includes two theory- and evidence-based components designed to work together synergistically: emotionally expressive writing and peer support writing. Building on evidence from a prior randomized control trial showing reductions in physical symptoms and distress in long-term transplant survivors with persistent survivorship problems, the Writing for Insight, Strength, and Ease (WISE) trial will evaluate the efficacy of expressive helping when used during transplant and in the early post-transplant period, when symptoms peak, and when intervention could prevent development of persistent symptoms.

**Methods:**

WISE is a multi-site, two-arm randomized controlled efficacy trial. Adult hematological cancer patients scheduled for a hematopoietic stem cell transplant will complete baseline measures and then, after hospitalization but prior to transplant, they will be randomized to complete either expressive helping or a time and attention “neutral writing” task. Both expressive helping and neutral writing involve four brief writing sessions, beginning immediately after randomization and ending approximately 4 weeks after hospital discharge. Measures of symptom burden (primary outcome), distress, health-related quality of life, and fatigue (secondary outcomes) will be administered in seven assessments coinciding with medically relevant time points from baseline and to a year post-intervention.

**Discussion:**

The steady and continuing increase in use of stem cell transplantation has created growing need for efficacious, accessible interventions to reduce the short- and long-term negative physical and psychosocial effects of this challenging but potentially life-saving treatment. Expressive helping is a psychological intervention that was designed to fill this gap. It has been shown to be efficacious in long-term transplant survivors but could have even greater impact if it is capable of reducing symptoms during and soon after transplant. The WISE study will evaluate these benefits in a rigorous randomized controlled trial.

**Trial registration:**

Clinicaltrial.govNCT03800758. Registered January 11, 2019

## Administrative information

Note: the numbers in curly brackets in this protocol refer to SPIRIT checklist item numbers. The order of the items has been modified to group similar items (see http://www.equator-network.org/reporting-guidelines/spirit-2013-statement-defining-standard-protocol-items-for-clinical-trials/).

**Table:** 

Title {1}	Efficacy of Expressive Helping in Adult Hematologic Cancer Patients Undergoing Stem Cell Transplant: Protocol for the Writing for Insight, Strength, and Ease (WISE) Study’s Two-Arm Randomized Controlled Trial.
Trial registration {2a and 2b}.	Clinicaltrial.gov: NCT03800758. Expressive Helping for Stem Cell Transplant Patients, registered January 11, 2019.
Protocol version {3}	**Issue Date**: 28 Aug 2020 **Protocol Amendment Number**: 14 **Author(s):** *C.R.; K.D.G*.
Funding {4}	This study is supported by the National Cancer Institute of the National Institutes of Health under award number R01CA223963.
Author details {5a}	^1^John Theurer Cancer Center, Hackensack University Medical Center, Hackensack, NJ, USA ^2^Lombardi Comprehensive Cancer Center, Georgetown University, Washington, DC, USA ^3^Department of Medical Social Sciences, Feinberg School of Medicine, Northwestern University, and the Robert H. Lurie Comprehensive Cancer Center, Northwestern University, Chicago, IL, USA ^4^Department of Psychology, William Paterson University, Wayne, NJ, USA ^5^Center for Community Health, Feinberg School of Medicine, Northwestern University, Chicago, IL, USA ^6^Departments of Psychology and Psychiatry/Biobehavioral Sciences and the Jonsson Comprehensive Cancer Center, University of California, Los Angeles, CA, USA ^7^Department of Psychology, Reykjavik University, Reykjavik, Iceland ^8^Department of Oncological Sciences, Icahn School of Medicine at Sinai School, New York, NY, USA
Name and contact information for the trial sponsor {5b}	National Cancer Institute of the National Institutes of Health, 9609 Medical Center Drive, Building 9609 MSC 9760, Bethesda, MD 20892-9760; Phone: 1-800-422-6237; E-mail: https://www.cancer.gov/contact/email-us
Role of sponsor {5c}	The content is solely the responsibility of the authors and does not necessarily represent the official views of the National Institutes of Health. The funding body had no role in study design, data collection and analysis, decision to publish, or preparation of the manuscript.

## Introduction

### Background and rationale {6a}

Hematopoietic stem cell transplant is used to treat hematological malignancies such as acute leukemia, lymphoma, and multiple myeloma, in addition to other malignant and non-malignant conditions. Nearly 25,000 transplants were conducted in the USA in 2018, continuing a steady increase that began in the 1980s [1]. This trend is expected to continue in coming years considering recent advances that allow this treatment to be used more broadly (e.g., for older patients, a greater number of racial and ethnic minority patients, and those with diagnoses not previously treated with a stem cell transplant) [1]. Although often capable of saving or substantially extending patients’ lives, transplant is associated with significant symptom burdens, namely physical and psychosocial burden due to medical factors (e.g., toxic effects of treatment, physical symptoms, risk for complications and late effects) and psychosocial stressors (e.g., threat to life, lengthy hospitalization and recovery period, financial burden, social and emotional isolation, disruption of personal and family roles) [2–6]. Symptom burden is especially troublesome during the acute phase of transplant, defined as the first 30 days [7]. Moreover, symptom intensity peaks during the lowest point of white blood cell count after high-dose chemotherapy, a period known as the “nadir” [7]. Symptom burden during hospitalization has been associated with higher post-traumatic stress disorder, depression, lower quality of life post-transplant, and higher mortality rates [8–15]. For a substantial proportion of recipients, symptoms become persistent, at times lasting for years after transplant [16–18]. As an adjunct to medical treatments and psychosocial resources targeting these issues, transplant recipients could benefit from efficacious, accessible psychological interventions capable of reducing the short- and long-term negative physical and psychosocial effects of this challenging but potentially life-saving treatment. To meet this need, we developed a psychological intervention called expressive helping [19]. It includes two theory- and evidence-based components designed to work synergistically: emotionally expressive writing and peer support writing. Patients first complete three brief, structured emotionally expressive writing sessions to develop insight into their experience before, during, and after transplant and to translate their thoughts and feelings about their transplant into language so that it can be more easily understood and communicated [20]. Building on these benefits, patients then complete a brief writing session to share their transplant experience, along with advice and encouragement, with the intention of providing peer support to other patients who are preparing for or undergoing transplant.

Meta-analyses and scientific reviews show that emotionally expressive writing improves physical and psychological outcomes in healthy and medically ill populations [21–23]. Some but not all studies of expressive writing in people diagnosed with cancer show similar benefits [24–26]; inconsistent findings may be due to methodological shortcomings (e.g., small sample sizes) and the need to consider moderators of effects of expressive writing in this population [27–29]. In our prior four-arm randomized controlled trial that included both expressive writing and expressive helping, we found limited evidence of benefits from expressive writing alone but clear evidence of benefits from expressive helping among long-term transplant survivors [19].

Even if expressive writing on its own yields limited or no improvements in outcomes such as physical symptoms, distress, and quality of life, its potential to help patients gain insight into their transplant experience and translate it into language creates a foundation for the second component of expressive helping: peer support writing. Growing evidence suggests that giving social support to others (e.g., peers) yields physical and psychosocial benefits for the support provider, including greater positive affect and self-concept [30–32], a stronger sense of social connection [30], lower psychological distress [31, 33], higher adherence to self-care [32, 34], and lower symptoms and other negative physical health outcomes [31, 33–35]. Although this evidence is mostly correlational, these findings are consistent with theories describing benefits of social support provision [36, 37] and the Helper Therapy Principle [38], which argues that giving support to others is more therapeutically beneficial than is receiving support. Initial research has begun to explore the biological mechanisms for the health benefits of giving support [39].

We believe that expressive writing facilitates patients’ ability to share their transplant experiences with peers by helping them gain insight into their transplant experience and helping them translate it into language, both of which can be expected to enhance their ability to communicate their experience with others. That is, expressive helping is based on the premise that there is a synergistic effect of completing emotionally expressive writing prior to peer support writing. This premise is consistent with effects we observed in our prior four-arm randomized controlled trial, which included a study arm in which participants completed peer support writing alone, without first completing expressive writing (i.e., writing as if talking to people preparing for transplant, sharing experiences, advice, and encouragement that might help other patients feel more prepared for transplant) [19]. This type of writing did not improve participants’ symptoms, distress, or quality of life.

In sum, our work has sought to translate the theoretically synergistic benefits of emotionally expressive writing and peer support writing into a brief intervention for cancer survivors who have undergone a stem cell transplant. As noted, we have demonstrated benefits of expressive helping in a four-arm, parallel groups, assessor-blinded randomized controlled trial. This trial compared expressive helping—completed in four brief structured writing sessions—with three other study conditions that included four sessions of the following: emotionally expressive writing with no peer support writing, peer support writing with no emotionally expressive writing, and neutral writing (control condition) [19]. Compared to the neutral writing control condition, expressive helping reduced physical and psychological symptoms in long-term transplant survivors who had undergone transplant in the prior 9 months to 3 years and who at baseline had moderate to severe persistent survivorship problems. Participants who completed emotionally expressive writing or peer support writing alone showed little or no benefit. We found no harms of expressive helping, which was determined to be a minimal risk intervention, and it was highly acceptable to participants.

## Objectives {7}

The overarching objective of this trial is to reduce patients’ symptoms during stem cell transplant and in the early post-transplant period, when patients likely are feeling their worst, and to prevent onset of persistent symptoms. Based on the findings of our randomized controlled trial with long-term transplant survivors and our goal of intervening earlier in the transplant experience, we adapted expressive helping so that it could be completed during the period that begins shortly after hospitalization for transplant and ends in the early post-transplant period.

Here, we describe the protocol for a two-arm randomized controlled trial called the Writing for Insight, Strength, and Ease (WISE) study, which will evaluate whether this adapted version of expressive helping, compared to a neutral writing (time and attention control) condition, improves physical and psychological symptoms among patients with hematological malignancies in the early post-transplant period and throughout the year after transplant. Primary hypotheses are that participants who complete expressive helping, compared to those who complete neutral writing, will demonstrate greater reduction in symptom severity from baseline to 3 months post-intervention (to evaluate effects of the intervention in the early post-transplant period) and from baseline to 12 months post-intervention (to evaluate effects of the intervention on development of persistent symptoms). Secondary hypotheses are that participants who complete expressive helping, compared to those who complete neutral writing, will demonstrate greater reduction in symptom severity during and after hospitalization for transplant, and more favorable changes in secondary outcomes (i.e., lower depressive symptoms, lower generalized and cancer-specific anxiety, better health-related quality of life, and lower fatigue) over time. We will also evaluate a select set of potential moderators of effects of expressive helping, including the severity of symptoms at baseline, actual or perceived constraints that limit participants’ expression of thoughts, feelings, or concerns to others (social constraints [40]) participant gender, race, and ethnicity.

## Trial design {8}

**Fig. 1:**
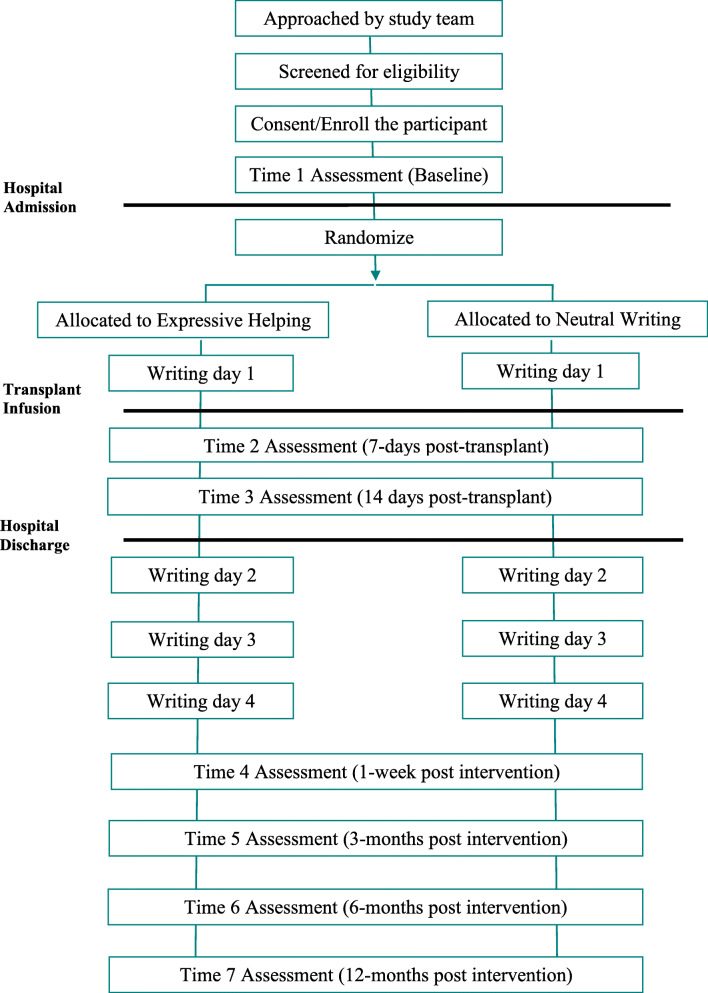
Study flow diagram

WISE is a two-arm, parallel groups, randomized controlled efficacy trial using 1:1 allocation to study arms. Figure 1 summarizes the study flow. Measures will be administered at seven points timed to coincide with key stem cell transplant events: a baseline assessment (time 1; prior to randomization and infusion), 7 days after infusion (time 2; approximately at the time of nadir, when symptoms are typically most severe), 14 days after infusion (time 3; approximately at the time of engraftment, when the infused blood-forming cells received at transplant begin to grow and make healthy blood cells), post-intervention (time 4; 1 week after the end of the intervention period), 3 months post-intervention (time 5; primary early endpoint), 6 months post-intervention (time 6), and 12 months post-intervention (time 7; primary long-term endpoint).

## Methods: participants, interventions, and outcomes

### Study setting {9}

We will recruit 310 participants across three transplant sites at academic medical hospitals in the USA, one in the Mid-Atlantic, one in the Northeast, and one in the Midwest.

### Eligibility criteria {10}

To be eligible, patients must be at least 18 years old, English proficient, and scheduled to have an allogeneic or autologous transplant to treat hematologic cancer. Patients will be excluded if they report that they are currently participating in a behavioral intervention aimed at reducing symptoms or improving quality of life, if they have cognitive or psychiatric impairment precluding ability to complete informed consent or study procedures (as determined by their physician or clinical team), if they have literacy limitations precluding completion of a writing study [41], or if their transplant is the first of two or more planned transplants being conducted as part of a tandem transplant approach. Patients about to receive a second/final transplant as part of a planned tandem transplant are eligible.

### Who will take informed consent? {26a}

Informed consent procedures will be conducted by trained staff members. Potential participants will be provided with a letter introducing the study, a study brochure, a handout summarizing the study activities, a copy of the informed consent and HIPAA authorization forms, and response cards to facilitate data collection through telephone interviews. A trained staff member will call or meet with potentially eligible patients to answer questions, determine interest in participating, and verify eligibility with a brief screening interview. Eligible patients interested in enrolling will then complete the informed consent procedures either in person (at a visit scheduled to coincide with a clinical appointment) or by telephone. Consent will occur prior to hospitalization or soon after hospitalization, before study procedures begin, and prior to the transplant infusion (“day 0”).

### Additional consent provisions for collection and use of participant data and biological specimens {26b}

There is no need for additional consent provisions.

## Interventions

### Overview

Using procedures and instructions adapted from our prior study with long-term transplant survivors [19], participants in both study arms will complete four structured writing sessions that begin during hospitalization and continue in the weeks following their hospitalization for stem cell transplant. Instructions for writing days 1, 2, and 3 ask participants to write for 20 min. On writing day 4, participants write for 20 min and have the option to continue writing for an additional 20 min. On all writing days, participants will be instructed not to worry about sentence structure, grammar, or punctuation. Participants will be asked to write continuously; if they run out of things to write, they will be instructed to repeat something they have already written.

Participants will complete writing sessions in a private, convenient location (e.g., hospital room, residence). As in our prior trial, writing sessions will be conducted by telephone. The procedures for the writing sessions are as follows: At the scheduled time, a trained staff member will call the participant to provide a brief, scripted introduction to the writing. Participants will then complete a pre-writing questionnaire while the staff member waits on the phone. The staff member will provide scripted instructions appropriate to the participant’s assigned study arm. The staff member will then ask the participant to start writing and end the call so the writing can begin, calling back after 20 min to end the writing. Participants interrupted for > 5 min during writing will be asked to continue writing for that length of time. Following the writing, participants complete a short post-writing questionnaire, after which the staff member will address questions or concerns and provide information about the next steps in the study, again using a scripted procedure.

For both study arms, the timing and writing instructions for the first three writing days allow participants to write about three meaningful stages of transplant soon after they complete each stage. Specifically, on writing day 1, completed after hospital admission and prior to transplant infusion, participants write about the time between finding out they needed a transplant and hospital admission; on writing day 2, completed 1 week after hospital discharge, they write about the time they spent in the hospital; and on writing day 3, completed 2 weeks after writing day 2 (3 weeks after hospital discharge), participants write about the experience of being out of the hospital after transplant. Specific writing instructions differ across study arms, as does the topic of writing day 4, which occurs 1 week after writing day 3.

### Explanation for the choice of comparators {6b}

The trial uses a neutral writing time and attention control condition, which enables us to isolate effects of theory- and evidence-based components of expressive helping while holding constant non-specific components, including contact with study staff and time spent on the writing [42].

#### Intervention description {11a}

*Expressive helping* combines expressive writing (writing days 1–3) and peer support writing (writing day 4), as already described. The concept of peer support is introduced in the instructions for writing day 1; they explain the following: (1) how transplant patients benefit from learning about other patients’ experiences, (2) our plan to develop a collection of peer support stories as a resource for patients, and (3) that writing days 1–3 are exercises in which participants write for themselves (i.e., not for sharing) to help them think about their transplant experience to prepare for writing the peer support story they can share in this resource. On writing days 1, 2, and 3, participants will receive emotionally expressive writing instructions based on Pennebaker’s standard protocol [20, 43, 44], adapted to ask participants to write about their deepest thoughts and feelings about the times before, during, and after transplant, respectively. On each of these writing days, participants are reminded that they are writing for themselves and that their writing will not be shared. Instructions for writing day 4 ask participants to write about any aspect of their transplant they think would help others prepare for this treatment, adding advice or encouragement if they wish. Participants are reminded that their day 4 writing will be a resource to help others feel more emotionally prepared for transplant, and they are asked to write as if speaking to someone preparing for transplant.

*Neutral writing* uses instructions we have used in past research [19, 27, 45]. The four neutral writing sessions will be completed using the same timing as sessions used in the expressive helping (intervention) arm. On writing days 1, 2, and 3, participants will receive instructions asking them to write a detailed factual account of their experiences before, during, and after transplant, respectively. Instructions for writing day 4 ask participants to write a factual account of their past week. This neutral writing task does not engage therapeutically active processes hypothesized to underlie benefits of expressive helping: instructions do not ask participants to explore their thoughts and feelings about transplant. All days of writing are framed as being for the participant; no mention is made of sharing the writing or of helping other patients.

Table 1 provides a summary of the writing instructions for both Expressive Helping and Neutral Writing.

**Table 1:** Summary of writing instructions for expressing helping and neutral writing

Writing day	Duration (min)	Expressive helping	Neutral writing
1	20	**Introduction** • Explain potential for people to benefit from writing about a stressful experience, including transplant. • Explain that many transplant recipients like the idea of helping others by sharing their experiences and advice, and that people preparing for transplant can benefit from knowing the full range of others’ experiences, even if their experience ends up being very different. Describe some possible benefits. • Explain that we are developing a collection of stories that will be a resource for people preparing for transplant and describe how the writing sessions will allow them to share their story and things that they wish someone had told them before transplant. • Remind them that their writing will not have their name on it and describe general rules for writing: (1) keep writing until time is up; (2) do not worry about spelling, sentence structure, or grammar; (3) delve deeply into transplant experience. **Specific instructions for day** • Write about what it was like when they found out they needed a transplant and how they prepared for it emotionally. • Ask them to really let go and explore their deepest thoughts and feelings about that time. • Remind them that the day’s writing will not be shared with anyone else—it is mainly to help them think about their experience and prepare to share it with others. • Reiterate rules about writing continuously and not worrying about spelling, sentence structure, or grammar.	**Introduction** • Explain potential for people to benefit from writing about a stressful experience, including transplant. • Remind them that their writing will not have their name on it and describe general rules for writing: (1) keep writing until time is up; (2) do not worry about spelling, sentence structure, or grammar. **Specific instructions for day** • Write detailed account of the facts regarding when they found out they needed a transplant and how they prepared for it before they went to the hospital. • Ask them to provide a detailed account of all that happened, focusing on facts and not thoughts and feelings about things that happened. • Remind them that the day’s writing will not be shared with others. • Reiterate rules about writing continuously and not worrying about spelling, sentence structure, or grammar.
2	20	**Specific instructions for day** • Write about what it was like for them during the time they spent in the hospital for their transplant. • Ask them to really let go and explore their deepest thoughts and feelings about that time. • Remind them that the day’s writing will not be shared with anyone else—it is mainly to help them think about their experience and prepare to share it with others. • Reiterate rules about writing continuously and not worrying about spelling, sentence structure, or grammar.	**Specific instructions for day** • Write detailed account of the facts regarding the time they spent in the hospital for their transplant. • Ask them to provide a detailed account of all that happened, focusing on facts and not thoughts and feelings about things that happened. • Remind them that the day’s writing will not be shared with others. • Reiterate rules about writing continuously and not worrying about spelling, sentence structure, or grammar.
3	20	**Specific instructions for day** • Write about what it was like for them in the time after they left the hospital. • Ask them to really let go and explore their deepest thoughts and feelings about that time. • Remind them that the day’s writing will not be shared with anyone else—it is mainly to help them think about their experience and prepare to share it with others. • Reiterate rules about writing continuously and not worrying about spelling, sentence structure, or grammar.	**Specific instructions for day** • Write detailed account of the facts regarding the time after they left the hospital. • Ask them to provide a detailed account of all that happened, focusing on facts and not thoughts and feelings about things that happened. • Remind them that the day’s writing will not be shared with others. • Reiterate rules about writing continuously and not worrying about spelling, sentence structure, or grammar.
4	20–40	**Specific instructions for day** • Describe how first three days of writing gave them a chance to think about their experiences and consider what they might want to share with people getting ready for transplant. • Remind them that the day’s writing will shared as part of a resource for people preparing for transplant, to help them feel more emotionally prepared for transplant and to help them cope with things that come up. • Ask them to write as if they are speaking to someone preparing for transplant. Reiterate day 1 information about how people benefit from knowing the full range of what other people experience, even if their experience ends up being very different. • Reiterate rules about writing continuously and not worrying about spelling, sentence structure, or grammar. • Tell them they will be able to write an extra 20 min if they desire.	**Specific instructions for day** • Write detailed account of the past week, including today. • Provide a justification for this task—to clarify details of their experience that are helpful to remember and appreciate how far they have come. • Ask them to provide a detailed account of all that happened, focusing on facts and not thoughts and feelings about things that happened. • Remind them that the day’s writing will not be shared with others. • Reiterate rules about writing continuously and not worrying about spelling, sentence structure, or grammar. • Tell them they will be able to write an extra 20 min if they desire.

### Criteria for discontinuing or modifying allocated interventions {11b}

This is a minimal risk study of a psychological intervention found to be safe in a prior trial; thus, we have not specified criteria for modifying allocated writing instructions. Participants may decide not to complete part or all of their assigned writing, and they may withdraw from the study at any time without penalty. We may also withdraw any participants who are unable to complete the required study tasks for any reason.

### Strategies to improve adherence to interventions {11c}

To improve adherence to the writing, we will schedule writing days within pre-determined windows to accommodate participants’ medical and life events. This flexibility is expected to optimize adherence while maintaining the rigor of the study, given evidence that the spacing of emotionally expressive writing sessions does not moderate its effects [22].

### Relevant concomitant care permitted or prohibited during the trial {11d}

We do not prohibit any concomitant medical care. Participants are ineligible for the study if they are currently participating in a behavioral intervention aimed at reducing symptoms or improving quality of life; however, they may receive psychological care or psychosocial resources during the study.

### Provisions for post-trial care {30}

We have not made provisions for ancillary or post-trial care, nor will we offer compensation for those who suffer harm from trial participation. As noted, this is a minimal risk intervention.

### Outcomes {12}

#### Primary outcomes and endpoints

Primary outcomes and endpoints are changes in symptom severity from time 1 to time 5 (early intervention effects) and from time 1 to time 7 (long-term intervention effects). These endpoints correspond with our goal to evaluate intervention effects both during the early post-transplant and in the later post-transplant (i.e., to evaluate whether the intervention prevents development of persistent symptoms).

#### Secondary outcomes and endpoints

Secondary outcomes and endpoints include changes in symptom severity from time 1 to other assessments occurring during and after hospitalization for transplant (times 2, 3, 4, and 6) and changes in depressive symptoms, generalized anxiety, cancer-specific anxiety, health-related quality of life, and fatigue from time 1 to times 2 through 7. Table 2 provides an overview of the patient completed assessments.

**Table 2:** Summary of patient-completed study assessments

Timing of assessment		Pre-transplant baseline	7 days post-transplant	14 days post-transplant	Intervention period	1 week post-intervention	3 months post-intervention	6 months post-intervention	12 months post-intervention
**Variable**	**Measure**	**Time 1**	**Time 2**	**Time 3**	**Writing Days**	**Time 4**	**Time 5**	**Time 6**	**Time 7**
**Primary outcome**									
**Symptom severity**	MDASI-BMT	X	X	X		X	X	X	X
**Secondary outcomes**									
**Depressive symptoms**	CES-D	X	X	X		X	X	X	X
**Anxiety**	GAD-7	X	X	X		X	X	X	X
	IES	X	X	X		X	X	X	X
**Health-related quality of life**	PROMIS Global Health	X				X	X	X	X
**Fatigue**	FACIT-Fatigue	X	X	X		X	X	X	X
**Potential covariates**									
**Demographics**		X							
**Sleep quality**	Pittsburgh Sleep Index	X				X	X	X	X
**Writing day measures**									
**Emotional and physiological arousal pre- and post-writing**	Standard items				X				
**Post-writing manipulation check**	Standard items				X				

*MDASI* MD Anderson Symptom Inventory-Bone Marrow Transplant, *CES-D* Center for Epidemiologic Studies Depression Scale, *GAD-7* 7-item Generalized Anxiety Disorder, *IES* Impact of Events Scale, *PROMIS* Patient Reported Outcomes Measurement Information System, *FACIT* Functional Assessment of Chronic Illness Therapy

### Participant timeline {13}

Figure 1 shows a diagram with the different phases of the study. Participants will be enrolled prior to hospital admission for transplant or after admission but prior to transplant (infusion of stem cells, or “day 0”). After enrollment, participants complete the time 1 (baseline) assessment. Like all study assessments, baseline will most often be administered by telephone or in-person by a trained study staff member using a script; however, participants can self-complete a paper version of the assessment (e.g., if the timing of medical procedures makes it difficult to schedule an interview). Time 1 may occur either prior to hospital admission or after hospital admission but prior to infusion. Writing day 1 will always occur after the baseline assessment and hospital admission, but prior to infusion. For writing day 1, a trained study staff member will call the participant, randomize the participant to a study arm, then use a script to complete writing day procedures, providing instructions appropriate to participants’ randomized assigned study arm. Subsequent assessments are completed by a staff member who is blind to the participant’s random assignment. Thus, a blinded staff member will call to administer the time 2 and time 3 assessments 7 days and 14 days after infusion, respectively. Next, writing day 2 will be completed 1 week after hospital discharge, with the caveat that the timing of hospital discharge varies and some participants may need to complete the time 3 assessment after hospital discharge. For these participants, writing day 2 may occur later than 1 week after hospital discharge; it will be scheduled to occur at least 3 days after the time 3 assessment to provide a time buffer between time 3 and participants’ completion of writing day 2. Writing days 3 and 4 occur 2 and 3 weeks after writing day 2, respectively. All writing sessions will use the telephone procedures described for time 1. The times 4–7 assessments will then be completed by a trained staff member who is blind to participants’ randomly assigned study arm; all occur after completion of the writing sessions. At the end of the time 7 assessment call, the staff member conducting the call will look up the participant’s random assignment then debrief the participant by describing the two study arms, the purpose of their assigned study arm, and the goals of the study. The staff member will also answer any participant questions.

Participants will be compensated for their time as they complete key study activities. They will receive a $25 gift card after completing the time 1, 4, 5, 6, and 7 assessments, totaling $125 for completion of all assessments.

### Sample size {14}

The primary hypotheses are that participants in the expressive helping study arm will demonstrate greater reduction in symptom severity from baseline (time 1) to 3 and 12 months post-intervention (times 5 and 7) compared to participants in the neutral writing study arm. Conservatively assuming 40% attrition at times 5 and 7, we will need to randomize 310 patients (155 per arm) to achieve 80% power to detect effect sizes of 0.46 (i.e., differences in mean change in symptom severity equal to 0.46 standard deviations) at both time points, at a significance level of 0.025 (by using Bonferroni adjustment for multiple testing).

### Recruitment {15}

We will recruit participants at three study sites. Monthly accrual goals will be set in proportion to the number of potentially eligible participants at each site. We will evaluate and troubleshoot accrual in weekly study meetings, adjusting recruitment protocols (e.g., the timing of approaching potential participants in the pre-transplant period) if necessary.

## Assignment of interventions: allocation

### Sequence generation {16a}

Participants will be randomized to either expressive helping or neutral writing with 1:1 allocation using a computer-generated block randomization. Randomization will be stratified by study site; sex, age (18–59 vs. ≥ 60), and transplant type (autologous, allogeneic) to distribute key medical factors such as conditioning toxicity and diagnosis across study arms.

### Concealment mechanism {16b}

Concealment will be accomplished electronically, in a data collection and management system implemented in Research Electronic Data Capture (REDCap) [46] and created, hosted, and maintained by the Georgetown University Lombardi Comprehensive Cancer Center’s Survey, Recruitment and Biospecimen Collection Shared Resource (SRBSR).

### Implementation {16c}

The SRBSR will generate the allocation sequence and implement it in the study’s REDCap system. Trained staff members will randomize participants during the writing day 1 call. Specifically, the staff member conducting the writing session will use the REDCap system to reveal a participant’s assigned study arm immediately prior to giving participants their writing day 1 instructions. .

## Assignment of interventions: blinding

### Who will be blinded {17a}

Staff conducting all follow-up interviews (times 2–7) will be blind to a participant’s randomly assigned study arm. Participants cannot be blinded; however, they will not be made aware of the study conditions nor the study hypotheses until they complete the final study assessment. We will ask participants not to discuss their writing with study staff.

### Procedure for unblinding if needed {17b}

Because this is a minimal risk trial, procedures for unblinding are not needed.

## Data collection and management

### Plans for assessment and collection of outcomes {18a}

#### Primary outcome measure

*Symptom severity* will be assessed with the M.D. Anderson Symptom Inventory-Bone Marrow Transplant (MDASI-BMT) [7, 47]. Using items from the core MDASI instrument, participants will rate the severity of 13 common symptoms experienced by cancer patients (e.g., pain, nausea); they will also rate the severity of five transplant-specific symptoms in the BMT module (e.g., mouth sores). Participants rate the worst severity of each symptom in the past 24 hours on a 0 to 10 scale (0 = not present to 10 = as bad as you can imagine). The mean of the responses is calculated to yield a symptom severity score in which higher scores indicate more severe symptoms. The MDASI-BMT has demonstrated good validity and internal reliability in past research [7].

#### Secondary outcome measures

*Depressive symptoms* will be assessed with the Center for Epidemiologic Studies Depression Scale (CES-D) [48], a 20-item measure in which respondents rate how often they have experienced symptoms associated with depression on a scale from 0 to 3 (0 = rarely or none of the time to 3 = most or almost all the time) in the past week. Scores are summed and range from 0 to 60, with high scores indicating greater depressive symptoms. A score of 16 or greater indicates possible clinical depression [48]. This measure has demonstrated good internal reliability in past research [49, 50].

Anxiety will be assessed in two ways. First, we will assess *generalized anxiety* with the Generalized Anxiety Disorder 7-item (GAD-7) scale [51], which measures the severity of symptoms associated with anxiety. Participants will rate the frequency with which they have experienced these symptoms in the past seven days on a scale from 0 = not at all to 3 = nearly every day. Scores are summed to create a scale that ranges from 0 to 21, with higher scores indicating greater generalized anxiety. Research has shown strong validity and reliability, and evidence suggests that a score of 5, 10, and 15 represent cutoffs for mild, moderate, and severe anxiety symptoms [51]. We will also assess *cancer-specific anxiety* using the Impact of Event Scale (IES) [52], which includes 15 items that assess difficulties (intrusive thoughts and feelings, avoidance) caused by a traumatic event (in this study, the diagnosis and transplant). Participants will rate how much they were distressed or bothered by each difficulty during the past seven days using a 5-point scale ranging from 0 = not at all to 4 = extremely. Responses are summed to produce a total score ranging from 0 to 60, where higher scores indicate greater symptomatology. This scale has shown strong validity and reliability in past research [53].

*Health-related quality of life* will be assessed with the PROMIS Global Health v1.2 [54], a validated 10-item measure that assesses aspects of physical and mental health functioning. Participants will rate items including their health, quality of life, physical and mental health, and satisfaction with social activities in the past seven days using a scale ranging from 5 = extremely to 1 = poor. Participants will also rate their ability to carry out their everyday physical activities on a scale from 5 = completely to 1 = not at all, their fatigue on a scale from 5 = none to 1 = very severe, and their pain on a scale from 0 to 10. Summed raw scores can be converted into *T*-score values. *T*-score distributions are standardized such that a 50 represents the average (mean) for the U.S. general population, and the standard deviation around that mean is 10 points. Prior research has shown that this scale has good internal reliability [55].

*Fatigue* will be assessed with the Functional Assessment of Chronic Illness Therapy-Fatigue (FACIT-F) scale [56], a 13-item measure that assesses quality of life related to fatigue in patients with cancer. Participants will answer how true each item was for them during the past seven days on a 0 to 4 scale (0 = not at all to 4 = very much). Eleven responses are reverse scored and responses are then averaged to yield a scale ranging from 0 to 52, in which higher scores indicate lower fatigue. The scale has good internal reliability [57, 58].

#### Potential covariates

*Sleep* during the past seven days will be assessed with the Pittsburg Sleep Quality Index [59], which assesses sleep habits and quality, including usual sleep and awake times and factors that may interfere with sleep. Seven component scores (subjective sleep quality, sleep latency, sleep duration, sleep efficiency, sleep disturbance, use of sleep medication, daytime dysfunction) are derived and summed to produce a global score. Global scores range from 0 to 21. Higher scores indicate worse sleep quality. Prior research in cancer patients has shown that this scale has good internal reliability and validity [60, 61].

*Demographic variables* will be self-reported and will include participants’ age, gender, marital status, race and ethnicity, education level, household income, employment status, and insurance status at baseline.

*Medical data* will be abstracted from participants’ electronic medical records. It will include primary diagnosis and disease stage, pre-transplant conditioning regimen, and donor type as well as complications and medical status occurring in the year following transplant (e.g., infections, graft versus host disease, relapse). We will record comorbidities using the Hematopoietic Cell Transplantation-specific Comorbidity Index [62].

#### Manipulation check measures

*Writing measures* include emotions and physiological arousal before and after writing, assessed with items from previous expressive writing research (e.g., [63]). Participants will rate eight symptoms including racing heart, upset stomach, headache, and dizziness and 10 emotions including proud, nervous, sad, and guilty on scale from 1 = *Not at all* to 5 = *A great deal*. In order to test if participants followed writing instructions (i.e., a manipulation check), we will use a 6-item post-writing manipulation check, including perceptions of how personal their writing was and how much they revealed their emotions on a scale from 1 = not at all to 5 = a great deal. If participants did follow the instructions, scores should be higher in the expressive helping group than the neutral writing group. Six additional items included after writing day 4 will assess how much participants provided information that would be helpful for others undergoing transplant and to what extent the study has been valuable and a helpful experience. We developed these items to serve as a manipulation check for the expressive helping intervention [19], because of its peer support component.

### Plans to promote participant retention and complete follow-up {18b}

We will use multiple strategies to ensure retention and complete follow up including minimizing the burden of assessments; building rapport with patients and their healthcare providers; collecting information needed to contact participants in multiple ways, with their consent; sending reminder letters; sending a newsletter between time 6 and 7 (the longest gap between assessments); providing simple handouts summarizing study activities and our contact information; and being flexible when scheduling follow up assessments. To enable intent-to-treat analyses, participants who do not complete the writing sessions will be encouraged to complete follow-up assessments. When possible, we will record participants’ reason for withdrawing from the study.

### Data management {19}

All data will be entered electronically into the study’s REDCap data collection and management system, which includes range checks for data values. In addition, we have procedures in place for checking for accurate data entry as well as daily back up of study data, data cleaning, and data coding.

### Confidentiality {27}

Only staff who require access to individually identifiable participant information for their roles will have access to it. During the trial, we will store study data downloaded from REDCap or gathered from other sources on a university-hosted, secure, Health Insurance Portability and Accountability Act (HIPAA)-compliant computer storage platform. We will not store identifiable participant information on laptop computers or other mobile computing hardware. Computers used to access data will be protected by a username and password that meet our institutions’ security requirements, and they will be protected with anti-virus software and scanned regularly for vulnerabilities. Paper forms collected from participants (e.g., surveys or writing materials) will be identified solely by participants’ confidential study identification number and stored in a locked filing cabinet in study offices.

### Plans for collection, laboratory evaluation, and storage of biological specimens for genetic or molecular analysis in this trial/future use {33}

This study will not collect biological specimens.

## Statistical methods

### Statistical methods for primary and secondary outcomes {20a}

The data will be collected at seven time points, as described above. Descriptive statistics will be calculated for each time point, overall and for subgroups of interest. These statistics will include means (standard deviations) or medians (interquartile range) for continuous variables, and counts (percentages) for categorical variables. Missing data issues will be addressed in sensitivity analyses using multiple imputation methods with 10 multiply imputed datasets [63]. To evaluate the efficacy of expressive helping, we will perform intent-to-treat analyses using linear mixed effects models—one model for each of the six outcomes (change in symptom severity, change in depressive symptoms, change in generalized anxiety, change in cancer-specific anxiety, change in health-related quality of life, and change in fatigue). We have a total of 36 outcome by time point combinations, of which two are considered primary (i.e., changes in symptom severity from baseline to 3 and 12 months post-intervention, respectively) and the remaining 34 are secondary. The linear mixed effects models will include a random effect for participant and the following fixed effects: randomization arm (expressive helping vs. neutral writing), time point (time 2 to time 7), randomization arm by time point interaction, the baseline value of the measure, study site, sex, age (18–59, ≥ 60), and transplant type (autologous, allogeneic). We will use these linear mixed effects models to perform hypothesis tests to compare the two study arms with regard to the means of the outcomes at each time point, and also provide estimated mean differences with corresponding 95% confidence intervals [64].

### Interim analyses {21b}

We do not plan to conduct interim analyses and we have not specified stopping guidelines.

### Methods for additional analyses (e.g., subgroup analyses) {20b}

We will explore whether the effects of expressive helping on outcomes differ according to symptoms at baseline (MDASI scores), social constraints, sex, race, and ethnicity. These analyses will evaluate the presence of two-way and three-way interaction terms involving the potential moderator, randomization arm (expressive helping vs. neutral writing), and timepoint (T2 to T7), using linear mixed effects models conducted in the same way as those described above.

### Methods in analysis to handle protocol non-adherence and any statistical methods to handle missing data {20c}

We will use several strategies to ensure that participants complete all four of their assigned writing days, including building rapport with patients and their healthcare providers, reminding them of upcoming writing days, and being flexible when scheduling writing days. However, we recognize that some non-adherence is unavoidable given participants’ medical treatment and health problems. Primary analyses will use an “intent-to-treat” approach, and missing data issues will be addressed in sensitivity analyses using multiple imputation methods with 10 multiply imputed datasets [65].

### Plans to give access to the full protocol, participant level-data, and statistical code {31c}

We will share our protocol, statistical code, and/or de-identified dataset with individuals who request access. Researchers who request the dataset will be asked to submit plans for analyses to avoid overlap with other analyses.

## Oversight and monitoring

### Composition of the coordinating center and trial steering committee {5d}

This trial will be overseen by an executive committee that includes the principal investigator at Northwestern University, site principal investigators at the other sites (one of whom is a transplant oncologist), and the project coordinator at Northwestern University. Data management is overseen by the Georgetown University Lombardi Comprehensive Cancer Center’s SRBSR. Georgetown University serves as the institutional review board (IRB) of record, and study activities at each site are also overseen by the site’s IRB.

### Composition of the data monitoring committee, its role, and reporting structure {21a}

This trial will not use a data monitoring committee, consistent with National Cancer Institute policy, which calls for monitoring that is commensurate with a trial’s degree of risk, size, and complexity. It will be monitored by its executive committee, which consists of the principal investigator, site principal investigators, and the project coordinator at Northwestern University, guided by monitoring and reporting requirements of the study’s IRB of record and site IRBs.

### Adverse event reporting and harms {22}

Study staff will monitor adverse events in their contacts with participants, participants’ responses to measures of distress, and participants’ writing from the writing sessions. They will report potentially adverse events to the executive committee, which will determine the necessary response and reporting, conferring with the IRB when necessary. Staff will implement reporting and management activities determined to be necessary. Serious adverse events and adverse events that are unexpected and related or possibly related to the study protocol will be documented in a safety report, which will provide a written account of the event, and reported to the relevant lead and site IRBs and agencies per currently approved institutional policies. Events that are either not related to participation in the study or that are expected and that do not occur at a frequency or severity indicates participants are at greater risk of harm than was previously known will be recorded and monitored by the study team and reported at the next annual IRB continuation review.

### Frequency and plans for auditing trial conduct {23}

We do not specify formal auditing plans, given the minimal risk nature of the trial. However, rigor and fidelity to the approved trial protocol are monitored through weekly meetings that include leadership and staff at all three sites, weekly meetings of staff engaged in enrollment and data collection, and monthly meetings of the full study team, including members of the executive committee.

### Plans for communicating important protocol amendments to relevant parties (e.g., trial participants, ethical committees) {25}

We will communicate important protocol amendments to the study team in weekly meetings, via email, and by updating study documentation of procedures and the protocol. Amendments that need to be communicated to participants will be communicated via letter. Communications to other relevant parties will occur in accordance with site policies.

## Dissemination plans {31a}

We will publish findings in peer-reviewed scientific journals and deposit publications into PubMedCentral per the NIH Public Access Policy. Data will be shared with the scientific community through presentations at local, regional, national, and international professional meetings. We will seek opportunities to share our findings with the public (e.g., through community events sponsored by our institutions). Participants will receive a newsletter after the completion of the study with an overview of the study results.

## Discussion

Our goal is to reduce symptoms and other negative outcomes in the growing population of cancer patients undergoing hematopoietic stem cell transplant, both in the early post-transplant period and in the later post-transplant period, when a substantial proportion of survivors have persistent physical and psychological symptoms. We will seek to achieve this goal with a low cost, easy-to-disseminate intervention that can be used as an adjunct to patients’ clinical care. The WISE study applies a rigorous randomized controlled design that builds on our prior trial with expressive helping and the evidence it yielded that the intervention can reduce physical and psychological symptoms in long-term transplant survivors with moderate to severe transplant survivorship symptoms [19]. Thus, this trial has the potential to extend our prior study’s evidence that transplant recipients can benefit from engaging in emotionally expressive writing paired with an activity that many are highly motivated to undertake—reaching out to help their peers be more prepared for transplant.

## Abbreviations

CES-D: Center for Epidemiologic Studies Depression Scale
FACIT: Functional Assessment of Chronic Illness Therapy
GAD-7: 7-item Generalized Anxiety Disorder
IES: Impact of Events Scale
IRB: Institutional Review Board
MDASI: MD Anderson Symptom Inventory-Bone Marrow Transplant
PROMIS: Patient Reported Outcomes Measurement Information System
REDCap: Research Electronic Data Capture
SRBSR: Georgetown University Lombardi Comprehensive Cancer Center’s Survey, Recruitment and Biospecimen Collection Shared Resource
